# Cut-off values for diagnosis of G6PD deficiency by flow cytometry in Thai population

**DOI:** 10.1007/s00277-022-04923-7

**Published:** 2022-07-15

**Authors:** Anchalee Thedsawad, Wanchai Wanachiwanawin, Orathai Taka, Chattree Hantaweepant

**Affiliations:** Division of Hematology, Department of Medicine, Faculty of Medicine Siriraj Hospital, Mahidol University, 2 Wanglang Road, Bangkoknoi, Bangkok, 10700 Thailand

**Keywords:** Intracellular G6PD activity, Bright cells, G6PD mutation, Quantitative MR, Multiplex amplification refractory mutation system-polymerase chain reaction (multiplex ARMS-PCR), Thai

## Abstract

In heterozygous females, X-inactivation causes a change in glucose-6-phosphate dehydrogenase (G6PD) activity from normal to deficient. Most G6PD screening tests are used to accurately diagnose hemizygous males, but they are less reliable for diagnosing heterozygous females. This study established flow cytometric cut-off values for screening of G6PD deficiency in hemizygous males and heterozygous or homozygous females. We studied 205 (125 females, 80 males) leftover blood samples from quantitative methemoglobin reduction (MR) screening. G6PD gene mutations determined by multiplex amplification refractory mutation system-polymerase chain reaction and direct DNA sequencing were used as the gold standard reference. Accuracy of the test, including the sensitivity, specificity, and positive and negative predictive values, was analyzed using MedCalc software. The optimal cut-off values for classification of %red blood cells with normal G6PD activity or %bright cells into homozygous normal, heterozygous, and homozygous deficiency in females were 85.4–100%, 6.3–85.3%, and 0–6.2%, respectively (sensitivity 93.2%, specificity 100%). The cut-offs for classification into hemizygous normal and hemizygous deficiency in males were 76.5–100% and 0–76.4%, respectively (sensitivity 100%, specificity 96.5%). Flow cytometry can be used to differentiate heterozygous females with intermediate phenotype from homozygous females, but cannot distinguish between heterozygous females with extreme phenotype and homozygous females. By flow cytometry, heterozygous and homozygous deficiency was detected in 29.6% and 3.2% of females, respectively. Among males, hemizygous deficiency was found in 31.3%. Flow cytometry can be used to screen patients with G6PD deficiency, and reliably and efficiently identify heterozygous and homozygous females, and hemizygous males based on cellular G6PD activity.

**Supplementary Information:**

The online version contains supplementary material available at 10.1007/s00277-022-04923-7.

## Introduction

Glucose-6-phosphate dehydrogenase (G6PD) is the first enzyme of the pentose phosphate pathway (PPP), and it converts nicotinamide adenine dinucleotide phosphate (NADP+) into reduced nicotinamide adenine dinucleotide phosphate (NADPH), which is essential for protection against oxidative stress in red blood cells (RBCs) [1]. The prevalence of G6PD deficiency in Thailand was reported to be 6.6–17% depending on the method of measurement, and whether the measurements were taken in endemic or non-endemic areas of malaria [2–4]. G6PD deficiency is transmitted by the X chromosome, which means that males are most often affected. Females are classified into three phenotypes, including homozygous normal, homozygous deficiency, and heterozygous for the G6PD gene [5]. In heterozygous females, X-inactivation causes variability in G6PD activity, which results from different proportions of 2 RBC populations (normal and deficient G6PD activity). Most G6PD screening tests can be used to clearly diagnose hemizygous males and homozygous-deficient females, but they are less reliable for diagnosing heterozygous females [6].

Several quantitative and qualitative assays are available for diagnosing G6PD deficiency, and most tests measure enzymatic activity in lysate from all RBCs, which reflects the overall G6PD activity in a blood sample [7, 8]. Semiquantitative assays using whole-cell lysate may classify heterozygous females as normal, even if they have residual enzymatic activity of 10–60% of normal levels. As a result, 50–70% of heterozygous females with partial G6PD deficiency can be missed [9, 10]. However, cytochemical staining of intracellular G6PD activity on intact RBCs facilitates detection of mosaic RBC populations in heterozygous females [8, 11, 12]. Flow cytometry is a method that produces a fluorescent readout of a classic methemoglobin reduction (MR) test for G6PD deficiency at the level of an individual RBC [11–13]. In the case of heterozygous females, both the populations of G6PD normal and G6PD-deficient RBCs can be observed by flow cytometry [7, 11, 12].

Therefore, this technique has higher diagnostic power than qualitative and quantitative MR for diagnosing G6PD deficiency in heterozygous females [8, 11, 12, 14]. The aims of this study were to establish flow cytometric cut-off values and normal values for screening of G6PD deficiency in hemizygous male and heterozygous or homozygous female individuals, to determine sensitivity and specificity of the test, and to study genetic heterogeneity of G6PD deficiency in the Thai population.

## Materials and methods

### Blood samples

A total of 205 blood samples (125 females, 80 males) from individuals aged over 18 years were obtained from leftover blood samples in citrate phosphate dextrose adenine (CPDA) for routine screening of G6PD deficiency at the Division of Hematology, Department of Medicine, Faculty of Medicine Siriraj Hospital, Mahidol University, Bangkok, Thailand. The routine method used to screen for G6PD deficiency at our center has historically been quantitative MR test. However and as a result of advances in technology and methods, the evaluation of intracellular G6PD activity in RBCs can be evaluated by flow cytometry, and G6PD genotyping can be performed using multiplex amplification refractory mutation system-polymerase chain reaction (multiplex ARMS-PCR) or direct DNA sequencing. In this study, molecular genotyping was used as the reference standard for assessing G6PD status. Hematologic parameters were measured using a Sysmex XN-3000 Automatic Hematology Analyzer (Sysmex Corporation, Kobe, Hyogo, Japan). The protocol for this study was approved by the Siriraj Institutional Review Board (SIRB) of the Faculty of Medicine Siriraj Hospital, Mahidol University, Bangkok, Thailand.

### Detection of G6PD deficiency by quantitative MR test

Methemoglobin (MetHb) is formed through the action of sodium nitrite on hemoglobin (Hb) of red blood cells. In the presence of Nile blue sulfate and glucose, MetHb is reduced to Hb via NADPH-dependent methemoglobin reductase. The rate of reduction is proportional to the G6PD activity of the cells. All sample analyses were performed in duplicate. Briefly, CPDA blood was centrifuged at 931*g* for 10 min. The plasma and buffy coat were removed. One volume of 1% (W/V) sodium nitrite (Sigma-Aldrich Corporation, St. Louis, MO, USA) was added to each volume of packed red cells, and incubated at room temperature (RT) for 20 min. Samples were washed 7–8 times with buffered saline solution and resuspended with 1.5 volumes of buffered saline solution. The 320 μl of nitrite-treated RBCs was combined with 100 μl of 0.3M glucose (Sigma-Aldrich) and 50 μl of 0.01% Nile blue sulfate (Sigma-Aldrich), and then incubated at 37°C for 3 h. For positive control, 1% (W/V) sodium nitrite was added. One hundred microliters of the incubated mixture was put into 10 ml of 0.02M phosphate buffer tube. After 10 min, 1 ml of hemolysate obtained from 0.02M phosphate buffer was transferred into 4 ml of 0.07M phosphate buffer tube and the optical density (OD) with spectrophotometer at 630 nm was read (OD1). Then, 20 μl of neutral sodium cyanide (10% (W/V) sodium cyanide (BDH, Poole, UK) and 12% (W/V) acetic acid (Merck, Darmstadt, Germany)) was added into 0.02M phosphate buffer tube which was stood for 5 min and OD at 630 nm was read (OD2). Finally, 20 μl of 20% potassium ferricyanide (Merck) was added into 0.07M phosphate buffer tube which was left for 5 min, followed by standing of 20 μl of neutral sodium cyanide for 5 min at RT and OD at 540 nm was read (OD3). The formula for calculating %MetHb and %Total Hb are as follows: %MetHb = [(OD-OD2)/*A*] × 100 and %Total Hb = [OD3/*B*] × 100, where *A* and *B* are the constants which are the slopes of standard curve obtained from the various known concentration of methemoglobin and total hemoglobin, respectively.

Percentage of methemoglobin reduction (%MR) from quantitative MR test was calculated from percent of MetHb within Total Hb. The cut-off value of %MR for G6PD deficiency of both male and female was more than 10%.

### Molecular studies

#### Detection of twelve common G6PD mutations by multiplex ARMS-PCR

Genomic DNA was extracted from a CPDA blood sample using a Gentra Puregene Blood Kit (QIAGEN, Hilden, Germany) according to the manufacturer’s protocol. Identification of the twelve common G6PD mutations was established using multiplex ARMS-PCR. Primers of 10 G6PD mutations were newly designed, including G6PD Gaohe (95A>G), G6PD Songklanagarind (196T>A), G6PD Quing Yan (392G>T), G6PD Mahidol (487G>A), G6PD Mediterranean (563C>T), G6PD Bangkok (825G>C), G6PD Viangchan (871G>A), G6PD Chinese-5 (1024C>T), G6PD Union (1360C>T), and G6PD Bangkoknoi (1502T>G) (Table S1). Primers for the other 2 mutations, namely G6PD Canton (1376G>T) and G6PD Kaiping (1388G>A), were previously published by Du et al. [15]. Multiplex ARMS-PCR was optimized to achieve all possible combinations of amplicons. PCR amplification was performed in a 30 μl reaction mix containing 100–300 ng of DNA, 1 pmol of each primer, 200 μM of each dNTP, 1.25 mM MgCl_2_, and 0.85 U of HotStar Taq (QIAGEN). The amplification conditions were processed in a Veriti 96-well Thermal Cycler (Applied Biosystems, Waltham, MA, USA), as follows: a first denaturation step at 96°C for 10 min, followed by 30 cycles with denaturation at 96°C for 1 min, annealing at 59–68°C for 1.30 min, extension at 72°C for 1 min, and a final extension of 10 min at 72°C. Amplicons were separated by electrophoresis in 2% agarose gel (SeaKem LE Agarose; Lonza Group AG, Basel, Switzerland), stained with safe dye (UltraPower DNA, Japan), and visualized under a blue light transilluminator (Vu-F Imager; Pop-Bio Imaging, Cambridge, UK).

#### Detection of G6PD gene mutations by direct DNA sequencing

Positive samples from quantitative MR test, but negative for the 12 known mutations by multiplex ARMS-PCR were subsequently tested using primer sequences for the 2nd–13th exon (the 1st exon cannot encode protein) and the 3′untranslated region (UTR) of the G6PD gene, as described by McDade et al. [16] and Chaowanathikhom et al. [17], respectively. Purification of the PCR products for preparation of the sequence template was performed using a GenepHlow Gel/PCR Kit (Geneaid, New Taipei City, Taiwan) followed by sequencing that was outsourced to Bio Basic (Singapore).

#### Detection of intracellular G6PD activity by flow cytometry

Flow cytometry was performed in duplicate in every blood sample to determine G6PD activity per individual RBC [12]. The assay is based on differences in the fluorescence capacities of oxyhemoglobin and methemoglobin. All oxyhemoglobin was converted to methemoglobin. Briefly, 100 μl of 5% hematocrit RBC suspension was combined with 100 μl of 0.125 M sodium nitrite (Sigma-Aldrich) and incubated at RT for 20 min. Samples were washed 3 times with phosphate-buffered saline (PBS) at 2095*g* for 3 min, and then resuspended in 100 μl PBS. The next step of the assay was to reduce methemoglobin to oxyhemoglobin in a NADPH-dependent and G6PD-dependent manner. After being washed, the then nitrite-treated RBCs were combined with 18 μl of 0.28M glucose (Sigma-Aldrich) and 6 μl of 0.01% Nile blue sulfate (Sigma-Aldrich), and then incubated at 37°C for 90 min with the lids of the Eppendorf tubes open. As the PPP produces NADPH, the Nile blue sulfate converts methemoglobin to oxyhemoglobin while consuming NADPH. After the completion of methemoglobin reduction, 2.5 μl of 0.4M potassium cyanide (Sigma-Aldrich) was added and incubated at RT for 5 min. Five microliters of each sample was added to 100 μl of 3% hydrogen peroxide (H_2_O_2_) in PBS, agitated vigorously by hand, and incubated at RT for 3 min. H_2_O_2_ reacts with oxyhemoglobin to produce a fluorescent product, but it does not react with methemoglobin. After incubation, the RBCs were washed 2 times with PBS at 2095*g* for 3 min. Samples were analyzed in duplicate using a BD LSRFortessa Flow Cytometer (BD Biosciences, San Jose, CA, USA). RBCs were incubated in the absence of glucose as a negative control for each sample.

The results of G6PD activity by flow cytometry are expressed as %bright cells. The normal-G6PD RBCs showed fluorescence, while G6PD-deficient RBCs showed no fluorescent signals.

### Statistical analysis

Data analysis was performed using SPSS statistical software (SPSS, Inc., Chicago, IL, USA) and MedCalc statistical software (MedCalc Software, Ltd., Ostend, Belgium). Receiver operating characteristic (ROC) curve analysis was used to identify the optimal cut-off point of %bright cells for defining heterozygous- or homozygous-deficient females, and hemizygous-deficient males. The optimal cut-off value was determined based on the Youden Index, and the sensitivity, specificity, positive predictive value (PPV), and negative predictive value (NPV). Analysis of the area under the ROC curve (AUC) was performed among the quantitative MR test, flow cytometry, and multiplex ARMS-PCR/direct DNA sequencing. Kolmogorov-Smirnov test or Shapiro-Wilk test was used to evaluate the data for normal distribution. Independent samples *t*-test or Mann-Whitney *U* test was used to compare CBC parameters and %bright cells among different subgroups. Categorical data analyses of quantitative MR, flow cytometry, and multiplex ARMS-PCR/direct DNA sequencing were performed using chi-square test. The kappa (*K*) coefficient was used to evaluate the level of agreement between two G6PD measurement methods. A *p*-value of less than 0.05 was considered to be statistically significant.

## Results

### Results of G6PD deficiency screening by quantitative MR test

Out of a total of 205 blood samples, 66 (32.2%) showed a positive result. G6PD deficiency was diagnosed in 41 of 125 (32.8%) in females, and in 25 of 80 (31.3%) in males.

### Results of G6PD mutation by multiplex ARMS-PCR or direct DNA sequencing

G6PD mutations were identified in 67 of 205 (32.7%) blood samples. G6PD mutations were identified in 44 of 125 (35.2%) female blood samples, including heterozygous deficiency (32.8%), homozygous deficiency (1.6%), and compound heterozygous deficiency (0.8%). G6PD mutations were identified in 23 of 80 (28.8%) male blood samples. One hundred and thirty-nine of 205 blood samples had normal G6PD activity by quantitative MR test, but 2.1% of those (3/139) was determined to have G6PD mutations by multiplex ARMS-PCR. Specifically, two heterozygous females were carrying G6PD Viangchan 871G>A, and one heterozygous female was carrying G6PD Gaohe 95A>G. Among the remaining 66 blood samples, G6PD mutations were detected in 63 samples by multiplex ARMS-PCR. Among the other 3 samples, 1 had G6PD mutations detected by direct DNA sequencing [female with compound heterozygous of 1311C>T and IVS11 T93C (G6PD1311T/93C)], but no mutations were detected in the two males. In this study, 8 G6PD mutations were detected from multiplex ARMS-PCR. The most common mutation was G6PD Viangchan 871G>A (9.8%), followed by G6PD Canton 1376G>T (8.3%), G6PD Kaiping 1388G>A (4.9%), G6PD Mahidol 487G>A (4.4%), G6PD Gaohe 95A>G (2.9%), and 0.5% each for G6PD Mediterranean 563C>T, G6PD Union 1360C>T, and G6PD Quing Yan 1024C>T (Table 1).

**Table 1:** Prevalence of G6PD variants in Thai population

G6PD variants	Nucleotide change	Male (*N*=80)		Female (*N*=125)				Total	% of total (*N*=205)
		Hemizygous		Heterozygous		Homozygous deficiency			
		*N*	%	*N*	%	*N*	%		
Viangchan	c.871 G>A	4	5.0	15	12.0	1	0.8	20	9.8
Canton	c.1376 G>T	10	12.5	7	5.6	0	0	17	8.3
Kaiping	c.1388 G>A	1	1.25	9	7.2	0	0	10	4.9
Mahidol	c.487 G>A	4	5.0	4	3.2	1	0.8	9	4.4
Gaohe	c.95 A>G	1	1.25	5	4.0	0	0	6	2.9
Mediterranean	c.563 C>T	1	1.25	0	0	0	0	1	0.5
Union	c.1360 C>T	1	1.25	0	0	0	0	1	0.5
Quing Yan	c.1024 C>T	1	1.25	0	0	0	0	1	0.5
Mahidol/Gaohe	c.487 G>A/c.95 A>G	0	0	0	0	1	0.8	1	0.5
Silent/IVS11-nt 93	c.1311 C>T /n.+93T>C	0	0	1	0.8	0	0	1	0.5
	Uncharacterized	2	2.5	0	0	0	0	2	0.9
	Total	25	31.25	41	32.8	3	2.4	69	33.7

*c*, codon; *n*, intron

### Identification of cut-off values of %bright cells by flow cytometry for females and males

**Fig. 1:**
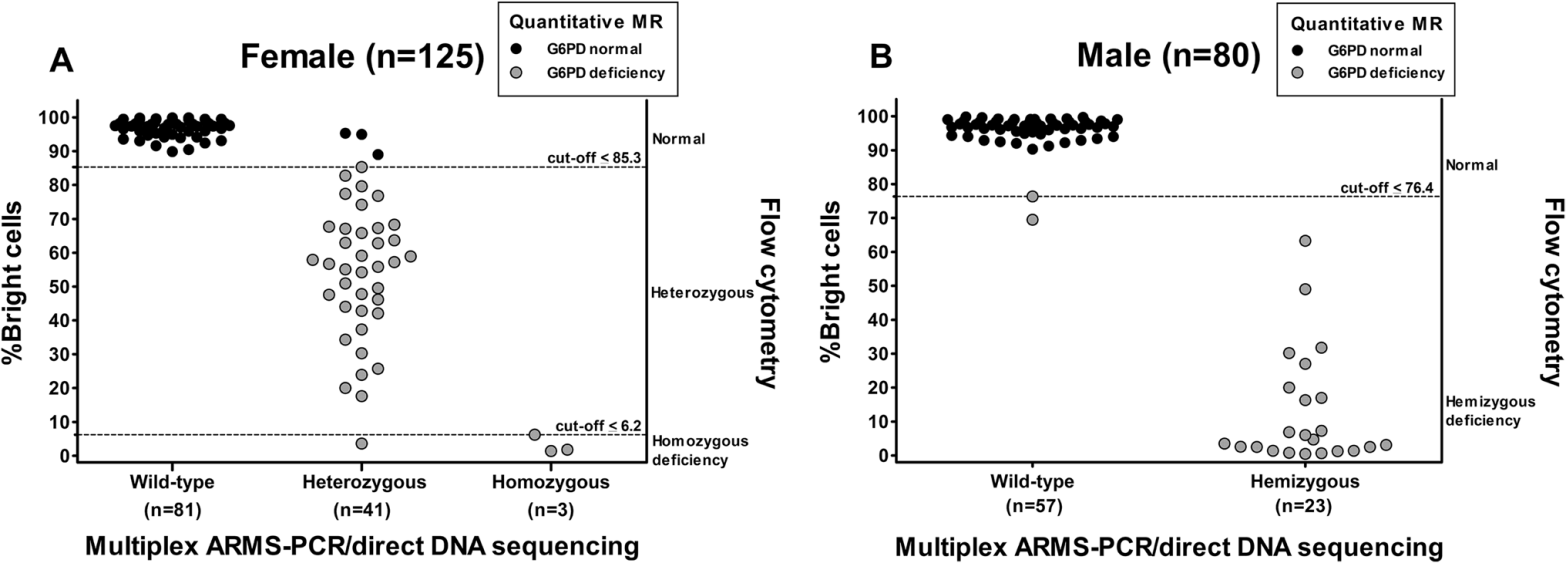
Dot plots show the distribution of %RBC with normal G6PD activity (%Bright cells) by genetic and quantitative MR test. Gray circles represent individuals who tested deficiency by quantitative MR. In females (**A**), dotted line at ≤6.2% bright cells represents the cut-off value of homozygous and dotted line at ≤85.3% bright cells represents the cut-off value of heterozygous as defined by multiplex ARMS-PCR/direct DNA sequencing. In males (**B**), dotted line at ≤76.4% bright cells represents the cut-off value of hemizygous as defined by multiplex ARMS-PCR/direct DNA sequencing

**Fig. 2:**
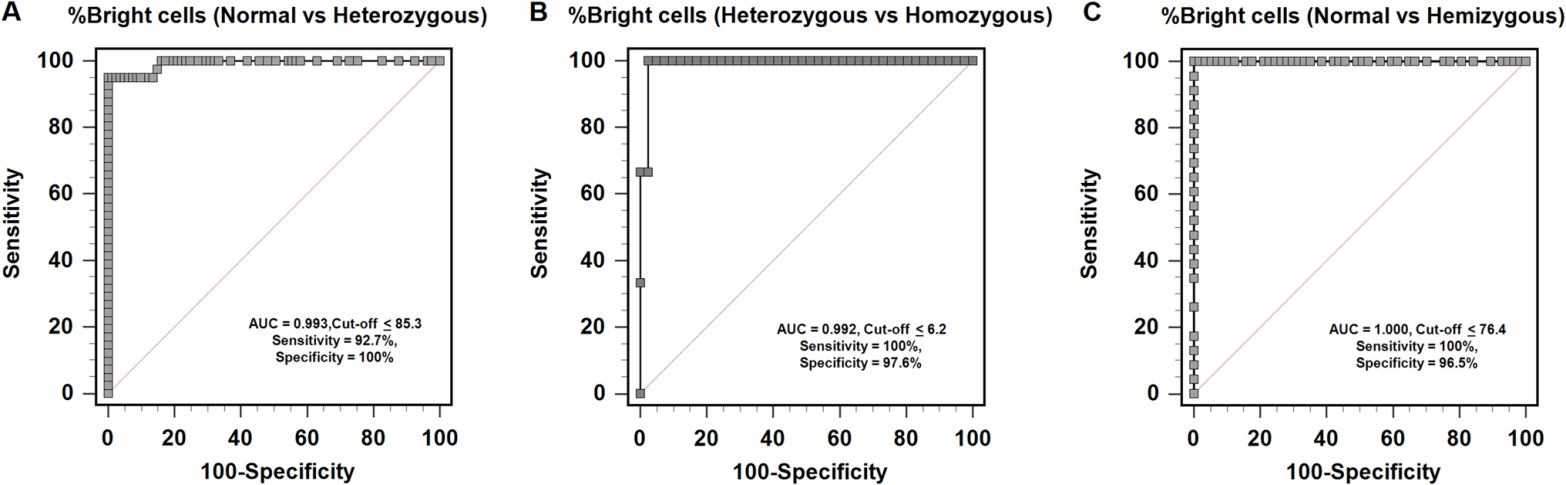
Graphs show receiver operating characteristic (ROC) curve analysis for cut-off values of %bright cells between normal female versus heterozygous female (**A**), between heterozygous female versus homozygous female (**B**), and between normal male versus hemizygous male (**C**)

A total of 205 blood samples were assessed using multiplex ARMS-PCR/direct DNA sequencing as the reference test. The results from genetic tests were categorized as G6PD normal or G6PD deficiency within both female and male samples for the purpose of identifying the optimal cut-off values of %bright cells from ROC curve analysis. Two male samples having positive results by quantitative MR, but undetectable G6PD mutation were categorized into the G6PD normal group, as shown in Fig. 1. As a result, the optimal cut-off values of %bright cells classified into homozygous normal, heterozygous, and homozygous deficiency in females were 85.4–100%, 6.3–85.3%, and 0–6.2%, respectively. These cut-off values could diagnose heterozygous females with and AUC of 0.993 (95% CI: 0.957–1.000), 92.7% sensitivity, 100% specificity, 100% PPV, and 98.6% NPV (Fig. 2A); and, diagnosed homozygous-deficient females with an AUC of 0.992 (95% confidence interval [CI]: 0.904–1.000), 100% sensitivity, 97.6% specificity, 88.5% PPV, and 100% NPV (Fig. 2B). Percent bright cells within the range of 76.5–100 and 0–76.4 were the cut-off values for discriminating hemizygous normal from hemizygous deficiency in males, respectively, with an AUC of 1.000 (95% CI: 0.955–1.000), 100% sensitivity, 96.5% specificity, 92% PPV, and 100% NPV (Fig. 2C).

After getting the cut-off values of %bright cells, they were used to interpret results in the same set of blood samples, as shown in Fig. 1. The results showed that there were 66 (32.2%) positive samples. Among the 125 female samples, 37 (29.6%) and 4 (3.2%) samples were classified into heterozygous and homozygous deficiency, respectively. Twenty-five (31.3%) of 80 male samples were classified into hemizygous deficiency.

### Comparison results of G6PD deficiency and level of agreement between quantitative MR, flow cytometry, and multiplex ARMS-PCR/direct DNA sequencing

The results of G6PD deficiency from quantitative MR and flow cytometry were compared with G6PD mutation results from multiplex ARMS-PCR/direct DNA sequencing. Quantitative MR and flow cytometry showed identical results (Table 2). The diagnostic performance was sensitivity 95.5%, specificity 98.6%, PPV 97.0%, and NPV 97.8%. When separated by gender, among female samples, the two tests also had the same sensitivity, specificity, PPV, and NPV (93.2%, 100%, 100%, and 96.4%, respectively). Within flow cytometry – flow cytometry demonstrated high sensitivity for detection of G6PD deficiency in females with homozygous, and in females with heterozygous deficiency (100% and 92.7%, respectively). Similarly, flow cytometry showed high specificity for the detection of G6PD deficiency in homozygous-deficient females and in the heterozygous group (97.6% and 100%, respectively) (*p*=0.943). In male samples, quantitative MR and flow cytometry showed the exact same sensitivity, specificity, PPV, and NPV (100%, 96.5%, 92.0%, and 100%, respectively; AUC: 1.000).

**Table 2:** The level of agreement in G6PD testing between multiplex ARMS-PCR/direct DNA sequencing and both flow cytometry and quantitative methemoglobin reduction (MR)

Test	Results	ARMS-PCR/direct DNA sequencing											
		All (*N*=205)				Female (*N* =125)				Male (*N* =80)			
		Positive (*N* =67)	Negative (*N* =138)	Kappa value	*p*	Positive (*N* =44)	Negative (*N* =81)	Kappa value	*p*	Positive (*N* =23)	Negative (*N* =57)	Kappa value	*p*
Flow cytometry	Deficiency (*N* =66)	64	2	0.944	<0.001	41	0	0.947	<0.001	23	2	0.941	<0.001
	Normal (*N* =139)	3	136			3	81			0	55		
Quantitative MR	Deficiency (*N* =66)	64	2	0.944	<0.001	41	0	0.947	<0.001	23	2	0.941	<0.001
	Normal (*N* =139)	3	136			3	81			0	55		

A *p*-value < 0.05 indicates statistical significance

The results of both quantitative MR and flow cytometry had almost perfect agreement with results of multiplex ARMS-PCR/direct DNA sequencing for detection of G6PD deficiency in all subjects, in females, and in males (*K*=0.944, 0.947, and 0.941, respectively). In addition, the results between quantitative MR and flow cytometry showed perfect agreement (*K*=1.000) for identification of G6PD deficiency in all samples, in female samples, and in male samples (Table 2).

### Comparison of laboratory data between G6PD normal and G6PD deficiency by quantitative MR and flow cytometry, including between each G6PD mutation group

Among all blood samples, there was no significant difference in hematological parameters between G6PD normal (139/205) and G6PD deficiency (66/205), including when separated by gender. When we compared hematologic parameters between each of the G6PD mutations, we found the MCV and MCH values of G6PD Viangchan 871G>A (78.3±14.3 and 24.4±5.2) to be significantly lower than G6PD Canton 1376G>T (91.7±13.5 and 28.7±3.4) and G6PD Gaohe 95A>G (95.1±7.7 and 29.9±1.4) (all respectively); however, no significant differences were found between the others. Among the 25 male samples with G6PD deficiency, the MCV and MCH values of G6PD Viangchan 871G>A (69.6±7.0 and 21.0±2.4) were significantly lower than those of G6PD Mahidol 487G>A (90.1±9.0 and 29.4±3.7) and G6PD Canton 1376G>T (93.8±13.5 and 29.5±3.3) (all respectively) because patients with G6PD Viangchan co-inherited with thalassemia disease. Among the 41 female samples with G6PD deficiency, no significant differences in hematologic parameters were observed between the different mutations.

**Fig. 3:**
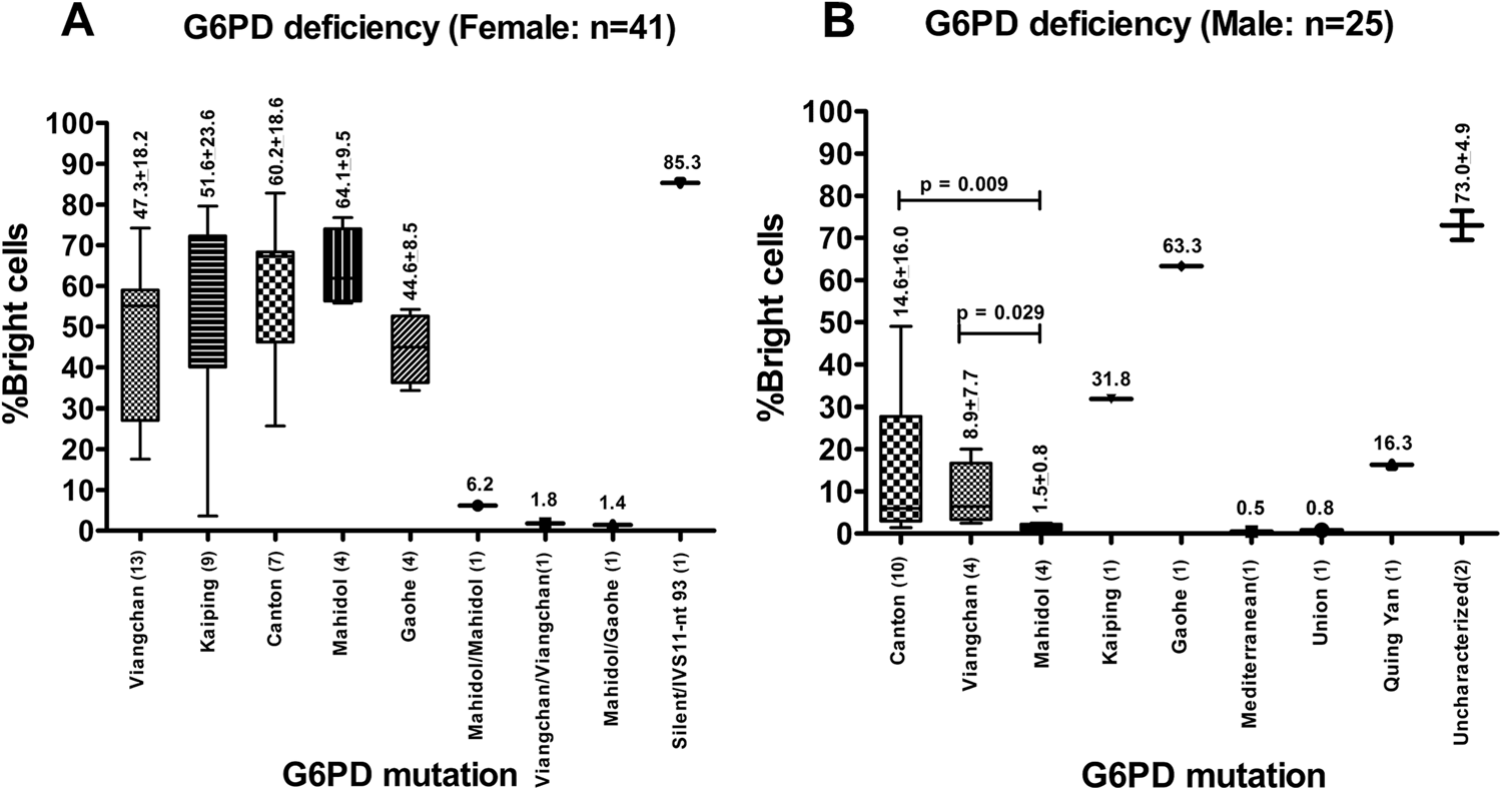
Box plots show the mean (plus/minus standard deviation) %RBC with normal G6PD activity (%Bright cells) for each of the G6PD mutations in females (**A**) and in males (**B**)

The %bright cells values between G6PD normal and G6PD deficiency in both female (97.5±2.4 and 49.5±22.7, respectively) and male samples (96.7±2.4 and 17.8±23.2, respectively) were significantly different (*p*<0.01). In addition, among males with G6PD deficiency, the %bright cells values of both G6PD Canton 1376G>T (14.6±16.0) and G6PD Viangchan 871G>A (8.9±7.7) were significantly higher than that of G6PD Mahidol 487G>A (1.5±0.8) (*p*=0.009 and *p*=0.029, respectively); however, no significant difference was found between each G6PD mutation among females, as shown in Fig. 3.

## Discussion

Using the flow cytometry and quantitative MR tests, the overall prevalence of G6PD deficiency was 32.2% in all, 32.8% in females, and 31.3% in males. By way of comparison, the prevalence by multiplex ARMS-PCR/direct DNA sequencing was 32.7% in all, 35.2% in females, and 28.8% in males. The higher prevalence in females by genetic testing may result from the increased ability of that test to detect heterozygous mutations with X-inactivation. However, the prevalence of G6PD deficiency in our study was comparatively higher than the rates reported from previous studies. Nantakomol et al. [9] reported that among 295 Thai healthy adults with unknown G6PD status, the prevalence of G6PD deficiency was 14.2%, among 15.8% females, and 8.9% males. Phompradit et al. [4] studied 317 Thai and Burmese individuals in malaria endemic areas and found a prevalence of G6PD deficiency of 6.6% in among all subjects, 4.4% in females, and 2.2% in males. These differences among studies may be due to differences among random samples in each study. In our study, the included samples were clinically suspected of having G6PD deficiency, so the prevalence was higher.

In our study, the most common mutation was G6PD Viangchan followed by G6PD Canton, G6PD Kaiping, and G6PD Mahidol. These four G6PD mutations accounted for 67–78% of G6PD deficiency mutations in Thai population [3, 4, 18]. The G6PD Gaohe, G6PD Union, G6PD Quing Yan, and G6PD Mediterranean mutations had low frequencies; however, they have been reported before in Thailand [3, 19, 20].

We found that a G6PD mutation in a female sample with G6PD deficiency was not detected by multiplex ARMS-PCR, but direct DNA sequencing showed the combination of 1311 mutation of C>T in exon 11, and 93T>C in intron 11. G6PD 1311T/93C is a special variant that does not make any change to the protein sequence of the G6PD enzyme, but the association of the two is the cause of reduced activity of the G6PD enzyme leading to clinical implications; however, the mechanism is unknown [21–23]. Moreover, two males with G6PD deficiency had a positive, but not very high %MR value (17.9% and 34.8%), and had an undetectable G6PD mutation by multiplex ARMS-PCR. These DNA samples were then subjected to further DNA sequence analysis of all coding regions, including in the 3′UTR of the G6PD gene, but no mutations were found. This may be due to mutations that occur in other regions of the gene that were not investigated in this study, such as mutation in the intronic flanking regions, or cryptic mutations in the promoter region [20, 24]. Another possible explanation is epigenetic mechanisms that influence gene transcription [25]. Toniolo et al. [26] reported G6PD expression to be associated with the methylation status of the promoter CpG island. Therefore, the two male samples that showed positive results by quantitative MR still cannot be excluded from the G6PD deficiency group in this study.

Our study showed the optimal cut-off values for classification of %bright cells into homozygous normal, heterozygous, and homozygous deficiency in female samples to be 85.4–100%, 6.3–85.3%, and 0–6.2%, respectively. These cut-off values are comparable to those reported by Kalnoky et al. [11] who reported ranges of 85–100%, 10–85%, and 0–10%, respectively. Our ranges of %bright cells for hemizygous normal and hemizygous deficiency in male samples were 76.5–100% and 0–76.4%, respectively. Our ranges were higher than the cut-off values reported by Kalnoky et al. [11] who reported %bright cell ranges of 50–100% and 0–50%, respectively. The discrepancy between these results may be due to different G6PD mutations and differences in the study populations. The cut-off values determined in this study were based on G6PD mutation types commonly found in Thai ethnic population. However, those reported by Kalnoky et al. [11] were based on G6PD mutation types found in African-American population, and in Burman/Karen ethnic groups from migrant populations residing along the Thailand-Myanmar border. Furthermore, we found one G6PD-deficient female who was interpreted as homozygous deficiency (3.6% bright cells) by flow cytometry, but only heterozygous G6PD Kaiping 1388G>A was identified. This finding may be explained by skewing of X-chromosome inactivation, which is defined as inactivation of an X-chromosome with a normal allele is favored over inactivation of an X-chromosome with a mutant allele. This phenomenon results in very low enzymatic activity similar to that of homozygous deficiency. In the random process of X-inactivation, the majority of heterozygous females have a proportion of G6PD-deficient red cells of about 50% (intermediate phenotype) [27]. However, few of them manifest skewing in favor of nearly normal red blood cells or nearly G6PD-deficient red blood cells, which is called an extreme (normal-like or homozygous-deficient-like) phenotype [28]. As a functional test, flow cytometry cannot distinguish between females with homozygous-deficient-like and homozygous-deficient phenotype. Thus, DNA analysis is still the gold standard method for detection of heterozygous females [29].

Apart from having similarly high sensitivity and specificity, the strengths of flow cytometry over quantitative MR are distinguishing between homozygous deficiency and heterozygous females (intermediate phenotype). The reason is that flow cytometry can detect G6PD activity at the single RBC level; thus, it can evaluate the actual proportion of normal and deficient-G6PD red cell populations [30]. In contrast, the quantitative MR test measures average G6PD activity in blood sample hemolysate containing a mixture of normal and G6PD-deficient RBCs. Furthermore, flow cytometry is a less time-consuming test and then it has faster turnaround time. It takes 3 h to complete with requiring manual operation for 1.5 h, whereas quantitative MR testing takes 6 h including 3 h of manual process. According to more technical advance of flow cytometry, we estimate its cost per one sample to be 20 USD, while the cost of quantitative MR test (in our institution) is 10 USD.

In conclusion, this study reported the optimal percentage of bright cells cut-off values by flow cytometry in both genders for interpreting G6PD deficiency in Thai population. Flow cytometry was demonstrated to be a useful method for screening patients for G6PD deficiency. This technique can be used to reliably and efficiently identify heterozygous and homozygous deficiency in females, and hemizygote in males based on cellular G6PD activity. However, female with normal G6PD activity still can be a carrier of G6PD deficiency and proper discrimination of heterozygous female only can be detected by DNA analysis.

## Supplementary information

ESM 1 (DOC 61 kb)
