# Supplementary material for: Cut-off values for diagnosis of G6PD deficiency by flow cytometry in Thai population
Source: Ann Hematol. 2022 Jul 15;101(10):2149–57. doi: 10.1007/s00277-022-04923-7 (PMC9463333; doi:10.1007/s00277-022-04923-7)
Supplement: Supplementary file 1 — (DOC 61 kb) [file 277_2022_4923_MOESM1_ESM.doc]

**Table S1** Primer information for multiplex ARMS-PCR of the G6PD mutation gene

| G6PD mutation | Primer (5’🡪3’ sequence) | Size (bp) |
| --- | --- | --- |
| Forward common (FC1) | GCC CTT GAA CCA GGT GAA CAG | - |
| G6PD Bangkok (825,G🡪C) (825FM) | GTC TGG TGG CCA TGG AGA A**C** | 776 |
| G6PD Viangchan (871,G🡪A) (871FM) | TGG CTT TCT CTC AGG TCA AG**A** | 284 |
| G6PD Chinese-5 (1024,C🡪T) (1024FM) | ACT TTT GCA GCC GTC GTC T | 129 |
| G6PD Bangkok (825,G🡪C) (825FN) | GTC TGG TGG CCA TGG AGA A**G** | 776 |
| G6PD Viangchan (871,G🡪A) (871FN) | TGG CTT TCT CTC AGG TCA AG**G** | 284 |
| G6PD Chinese-5 (1024,C🡪T) (1024FN) | ACT TTT GCA GCC GTC GTC **C** | 129 |
| Reverse common (RC1) | GTG AGT GTC TCA GTG GGA GC | 873 |
| Forward common (FC2) | GAC CTG ACC TAC GGC AAC AGA TAC | - |
| G6PD Union (1360,C🡪T) (1360RM) | AGC TGG GCC TCA CCT GC**A** | 220 |
| G6PD Canton (1376,G🡪T) (1376RM)^a^ | TGA AAA TAC GCC AGG CCT CA**A** | 344 |
| G6PD Kaiping (1388,G🡪A) (1388RM) ^a^ | GGT GCA GCA GTG GGG TGA AAT TA**T** | 359 |
| G6PD Bangkoknoi (1502, T🡪G) (1502RM) | CTT GTA GGT GCC CTC ATA CTG G**C** | 569 |
| G6PD Union (1360,C🡪T) (1360RN) | AGC TGG GCC TCA CCT GC**G** | 220 |
| G6PD Canton (1376,G🡪T) (1376RN) ^a^ | TGA AAA TAC GCC AGG CCT CA**C** | 344 |
| G6PD Kaiping (1388,G🡪A) (1388RN) ^a^ | GGT GCA GCA GTG GGG TGA AAA TA**C** | 359 |
| G6PD Bangkoknoi (1502, T🡪G) (1502RN) | CTT GTA GGT GCC CTC ATA CTG G**A** | 569 |
| Reverse common (RC2) | GAA TGT AGC TGG GCT CGG GTA G | 764 |
| Forward common (FC3) | CAG CAT GCC AGC AAT GCC AC | - |
| G6PD Mahidol (487,G🡪A) (487FM) | TCC GGG CTC CCA GCA GA**A** | 511 |
| G6PD Mediterranean (563,C🡪T) (563FM) | GAC CGG CTG TCC AAC CAC ATA T**T** | 440 |
| G6PD Mahidol (487,G🡪A) (487FN) | TCC GGG CTC CCA GCA GA**G** | 511 |
| G6PD Mediterranean (563,C🡪T) (563FN) | GAC CGG CTG TCC AAC CAC ATA T**C** | 440 |
| Reverse common (RC3) | GCC AGC CTC CCA GGA GAG A | 715 |
| Forward common (FC4) | CGT TCA CAA GGA GTG ATT TGG | - |
| G6PD Gaohe (95,A🡪G) (95RM) | GCA CCC ATG ATG ATG AAT ATG **C** | 256 |
| G6PD Gaohe (95,A🡪G) (95FN) | CCT TCC ATC AGT CGG ATA CAC **A** | 557 |
| Reverse common (RC4) | TAG GCA CAG GGT TCA GAA ACG | 770 |
| Forward common (FC5) | GCA GGA GAT GCG TTT ATG TC | - |
| G6PD Songklanagarind (196,T🡪A) (196RN) | GGC ATA GCC CAT GAT GA**A** | 401 |
| G6PD Songklanagarind (196,T🡪A) (196FM) | CTT CTG CCC GAA AAC ACC **A** | 368 |
| Reverse common (RC5) | CCT GGG AGA AAT ACA CTG GAG | 733 |
| Forward common (FC6) | CTG GAT GTG CAG AGC TGC TAA G | - |
| G6PD Quing Yan (392,G🡪T) (392RM) | AGG CGG TTG GCC TGT GAC **A** | 323 |
| G6PD Quing Yan (392,G🡪T) (392FN) | GAT GCC CTC CAC CTG GG**G** | 272 |
| Reverse common (RC6) | GTG TTT CGT GGA GCA ACG CTG | 560 |

^a^As reported by Du et al. [15]
